# Role of Histone Acetylation in the Stimulatory Effect of Valproic Acid on Vascular Endothelial Tissue-Type Plasminogen Activator Expression

**DOI:** 10.1371/journal.pone.0031573

**Published:** 2012-02-20

**Authors:** Pia Larsson, Erik Ulfhammer, Mia Magnusson, Niklas Bergh, Sebastian Lunke, Assam El-Osta, Robert L. Medcalf, Per-Arne Svensson, Lena Karlsson, Sverker Jern

**Affiliations:** 1 The Wallenberg Laboratory for Cardiovascular Research, Institute of Medicine, The Sahlgrenska Academy, University of Gothenburg, Gothenburg, Sweden; 2 Epigenetics in Human Health and Disease Laboratory, Baker IDI Heart and Diabetes Institute, The Alfred Medical Research and Education Precinct, Melbourne, Victoria, Australia; 3 Australian Centre for Blood Diseases, The Alfred Medical Research and Education Point, Monash University, Melbourne, Victoria, Australia; 4 Sahlgrenska Center for Cardiovascular and Metabolic Research, Department of Molecular and Clinical Medicine, Institute of Medicine, The Sahlgrenska Academy, University of Gothenburg, Gothenburg, Sweden; Leiden University Medical Center, Netherlands

## Abstract

**Aims:**

Stimulated release of tissue-type plasminogen activator (t-PA) is pivotal for an intravascular fibrinolytic response and protects the circulation from occluding thrombosis. Hence, an impaired t-PA production is associated with increased risk for atherothrombotic events. A pharmacological means to stimulate the production of this enzyme may thus be desirable. We investigated if the anti-epileptic drug valproic acid (VPA) is capable of enhancing t-PA expression *in vitro* in vascular endothelial cells, and further examined if its histone deacetylase (HDAC)-inhibitory activity is of importance for regulating t-PA expression.

**Methods and Results:**

Human endothelial cells were exposed to valproic acid and t-PA mRNA and protein levels were quantified. Potential changes in histone acetylation status globally and at the t-PA promoter were examined by western blot and chromatin immunoprecipitation. Valproic acid dose-dependently stimulated t-PA mRNA and protein expression in endothelial cells reaching a 2–4-fold increase at clinically relevant concentrations and 10-fold increase at maximal concentrations. Transcription profiling analysis revealed that t-PA is selectively targeted by this agent. Augmented histone acetylation was detected at the t-PA transcription start site, and an attenuated VPA-response was observed with siRNA knock of HDAC3, HDAC5 and HDAC7.

**Conclusions:**

Valproic acid induces t-PA expression in cultured endothelial cells, and this is associated with increased histone acetylation at the t-PA promoter. Given the apparent potency of valproic acid in stimulating t-PA expression *in vitro* this substance may be a candidate for pharmacological modulation of endogenous fibrinolysis in man.

## Introduction

Myocardial infarctions and a substantial part of ischemic strokes are caused by intravascular clot formation. When a clotting process is initiated in an otherwise healthy blood vessel, the surrounding endothelium is activated and releases large amounts of the fibrinolytic enzyme tissue-type plasminogen activator (t-PA) causing the clot to dissolve. Recent data from the Framingham Heart Study support the hypothesis that an impaired t-PA release may increase the risk for permanent flow-arresting thrombi, as the t-PA enhancer −7,351C/T polymorphism (generating a low-secretor phenotype) was found to be associated with a more than 3-fold adjusted increased risk for myocardial infarction [1]. A similar association was previously reported by us [2]. This indicates that in case of an impaired endogenous fibrinolysis due to a reduced capacity for t-PA release, intravascular thrombus formation may propagate and lead to arterial occlusion and tissue infarction. In line with this hypothesis, t-PA release has been found to be defective in certain conditions associated with increased risk of thrombotic events, such as hypertension [3], [4], [5], obesity [6] and coronary atherosclerosis [7], [8].

Our group has previously demonstrated that it is possible to restore a suboptimal t-PA response by treating the underlying condition [5]. However, it is desirable to find a broader pharmacological tool to stimulate endogenous local fibrinolysis also in cases when the patho-physiological mechanism causing the attenuated t-PA release is unknown or inaccessible for intervention. The fact that t-PA expression has been reported to be powerfully up-regulated by the classical histone deacetylase inhibitors (HDACi) butyrate and Trichostatin A (TSA) [9], [10], as well as to the newer HDACi MS-275 [11], suggests that the t-PA gene could be sensitive to changes in histone acetylation status. Post-translational acetylation status of histone proteins is determined by the concerted action of two families of enzymes, the histone acetyl transferases (HATs) which catalyze the transfer of an acetyl group to lysine residues of the histone tails, and histone deacetylases (HDACs) which catalyze the removal of subsequent acetyl groups. The combined action of these two groups of enzymes results in a dynamic regulation of the acetylation status of histones, which influence the transcriptional competence of chromatin (reviewed in [12]). Inhibition of HDACs is considered to prevent histone deacetylation hence shifting histone acetylation status in favor of a more acetylated and permissive chromatin state.

Valproic acid (valproate, 2-propylpentanoic acid, VPA) is a clinically available anti-epileptic and mood-stabilizing drug which is also suggested to be an HDAC inhibitor in several transformed cell lines [13], [14] as well as primary cells including endothelial cells [15]. In this study, we investigated the hypothesis that VPA functions as an HDAC inhibitor in endothelial cells and that HDAC inhibition by VPA could increase t-PA production *in vitro*. If so, VPA may be a promising candidate for modulation of local endogenous fibrinolytic capacity *in vivo*.

## Materials and Methods

### Chemicals

Na-valproate was purchased from Sigma-Aldrich (St Louis, MO, USA) and stock solutions (0.3 M) were prepared in complete endothelial cell culture medium (EGM-2, Lonza, Basel, Switzerland) and stored at −70°C. Valpromide (2,2-Di-n-propylacetamide) was ordered from Alfa Aesar (Karlsruhe, Germany) and stock solutions (1.5 M) were prepared in DMSO and stored at −20°C. The compounds were diluted in complete endothelial cell culture medium immediately before use.

### Cell culture and experimental design

The investigation conformed to the principles outlined in the Declaration of Helsinki for the use of human tissues, and the use of cells from human umbilical cords was approved by the Regional Ethics Review Board of Gothenburg (approval reference number 449–93). Verbal informed consent was obtained from the donors regarding the use of umbilical cord cells for research purposes. Given that the study material was non-identifiable, written consent was uncalled for and this consent procedure was approved by the ethical review board. Human umbilical vein endothelial cells (HUVECs) were prepared by collagenase treatment [16] of fresh umbilical cords obtained from the maternity ward of the Sahlgrenska University hospital. Cells were cultured in EGM-2 medium and all experiments were performed in passages 1 or 2. Human coronary artery endothelial cells (HCAEC) were purchased from Lonza and cultured in EGM-2 complete medium supplemented with fetal bovine serum to a total serum content of 5%. HCAEC were used in passage 5. Confluent HUVECs or HCAECs were exposed to optimal concentrations of VPA in complete medium. After incubation with the substance for up to 72 h (with fresh medium and inhibitors added every 24 h), cells and conditioned media were harvested. Cell cultures were performed in duplicate and all experiments (except HDAC9 siRNA experiments, n = 2) were performed on cells from a minimum of 3 individuals.

### Real-Time RT-PCR

Total RNA was prepared using RNeasy Mini RNA kit (Qiagen, Hilden, Germany) and genomic DNA was removed using RNase-free DNase I set (Qiagen). Levels of t-PA mRNA were analyzed with real-time RT-PCR, performed on an Applied Biosystems 7500 Fast Real-Time PCR System using cDNA and Taqman reagents obtained from Applied Biosystems (Foster City, CA, USA). Hypoxanthine phosphoribosyl transferase (HPRT) was used as endogenous internal standard, except in HDAC7 siRNA experiments where Beta-glucuronidase (GUSB) was employed. The primer sequences were as follows: t-PA Fp: 5′-GGC CTT GTC TCC TTT CTA TTC G-3′, t-PA Rp: 5′-AGC GGC TGG ATG GGT ACA G-3′, and t-PA probe 5′-TGA CAT GAG CCT CCT TCA GCC GCT-3′ The t-PA probe was dual-labeled with 5′-reporter dye FAM (6-carboxy-fluorescein) and 3′-quencher dye TAMRA (6-carboxy-tetramethyl-rhodamine). HPRT and GUSB transcripts were detected using Gene Expression Assays Hs99999909_m1 and Hs99999908_m1 (Applied Biosystems), respectively. Knock-down of class I, IIa and IV HDAC mRNAs were confirmed with the following Gene Expression Assays: Hs02621185_s1 (HDAC1), Hs00231032_m1 (HDAC2), Hs00187320_m1 (HDAC3), Hs00954353_g1 (HDAC8), Hs01041648_m1 (HDAC4), Hs00608366_m1 (HDAC5), Hs00248789_m1 (HDAC7), Hs00206843_m1 (HDAC9) and Hs00227335_m1 (HDAC11). u-PA and von Willebrand factor mRNA levels were detected using Gene Expression Assay Hs00170182_m1 and Hs00169795_m1 respectively, and PAI-1 mRNA was detected using the primers PAI-1 Fp 5′-GGC TGA CTT CAC GAG TCT TTC A-3′, Rp 5′-TTC ACT TTC TGC AGC GCC T-3′ and FAM/TAMRA labeled probe 5′-ACC AAG AGC CTC TCC ACG TCG CG-3′.

### Enzyme-linked immunosorbent assay

Endothelial cells in culture are known to constitutively secrete the majority of synthesized t-PA [17], making conditioned media a suitable source for quantification of t-PA protein. Conditioned medium from cell cultures was collected, centrifuged (10 000× g, 10 min, 4°C) to remove cell debris, transferred to fresh tubes and stored at −70°C. Concentrations of t-PA antigen in conditioned media were determined using the commercially available TriniLize t-PA antigen ELISA (Trinity Biotech, Bray, Ireland) according to manufacturer's protocol.

### Western blot analysis

Cells were harvested in Laemmli sample buffer with 5% β-merkaptoethanol, sonicated and boiled before being resolved on a pre-cast 10–20% Tris-Glycine gel (Lonza) and transferred by electro blotting onto polyvinylidene fluoride membranes. Membranes were incubated with primary antibodies to pan acetylated H3 (#39139) and acetylated H4 (#39243) (both from Active Motif, Carlsbad, CA, USA) as well as to total histone H3 (#ab1791, AbCam, Cambridge, UK) and H4 (#05-858, Millipore, Billerica, MA, USA). Proteins were detected according to manufacturer's protocols and visualized using chemiluminescence (SuperSignal, Pierce Biotechnology, Rockford, IL, USA).

### Chromatin Immunoprecipitation Assay

HUVECs were grown on 15 cm plates until confluent and then stimulated with VPA for 24 h. After washing, cross-linking (1% formaldehyde in PBS, 5 min), glycine quenching, and further washing steps, cells were lysed in 300 µl of lysis buffer (1% SDS, 10 mM EDTA, 50 mM Tris-HCl pH 8,0) supplemented with protease inhibitors and immediately sonicated to shear chromatin to a length of approximately 100–500 bp (as determined by capillary electrophoresis on an Agilent 2100 bioanalyzer) using a Diagenode Biorupture (30 min of 30 s on/off at 4°C). Insoluble material was removed by centrifugation (12 000× g, 10 min) and chromatin concentration was determined using the Quant-iT dsDNA BR kit and Qubit fluorometer (Invitrogen Life Technologies, Carlsbad, CA, USA). Immunoprecipitation was performed on 1 µg of sheared chromatin, corresponding to approximately 5×10^5^ cells per IP. Chromatin was diluted to 500 µl in dilution buffer (0.01% SDS, 1.1% Triton X-100, 1.2 mM EDTA, 16.7 mM Tris-HCl pH 8,0, 167 mM NaCl) and pre-cleared by the addition of 20 µl Protein A-coupled magnetic beads (Invitrogen) for 2 h on constant rotation. Four µg of each antibody and 20 µl of protein A beads were pre-incubated for 2 h before the addition of the pre-cleared chromatin and the reactions were left over night at 4°C on a rotating platform. The antibodies used were the same as for western blot detecting pan acetylated histone H3 (K9, 14, 18, 23, and 27) or pan acetylated histone H4 (K5, 8, 12, 16). For detection of mono-lysine acetylation the following antibodies from Active Motif were used: acH3K9: #39137, acH3K14: #39697, acH3K18: #39587, acH3K23: #39131, acH3K27: #39133, acH4K5: #39169, acH4K12: #39165, and acH4K16: #39167. The antibody for acH4K8 (07-328) was purchased from Millipore. A no-antibody control reaction was included in each run. After extensive washing, captured DNA was eluted from the beads by proteinase K digestion (Sigma-Aldrich) and purified using spin column purification (Nucleospin Extract II, Macherey-Nagel, Düren, Germany). Isolated DNA fragments were quantified using real-time PCR with SYBR Green detection and the following primers spanning (−46 to +92) the t-PA major transcription initiation site (TIS) [18] (+66 to +203 according to the minor TIS): Forward primer 5′-ACCCCCTGCCTGGAAACTTA-3′ and reverse primer 5′-GGTACAGAAACCCGACCTACCA-3′. Levels of precipitated DNA are displayed as percentage of input (non-precipitated control) corrected for background-(No antibody) precipitated DNA.

### Short interfering RNA transfection

Short interfering RNA (siRNA) specific for class I HDACs (HDAC1, HDAC2, HDAC3 and HDAC8), class IIa HDACs (HDAC4, HDAC5, HDAC7 and HDAC9) and class IV HDAC (HDAC11) were obtained from Dharmacon (Thermo Fisher Scientific, Lafayette, CO, US). The following ON-TARGETplus SMART pool siRNA sets were used: HDAC1; L-003493, HDAC2; L-003495, HDAC3; L-003496, HDAC8; L-003500, HDAC4; L-003497, HDAC5; L-003498, HDAC7; L-009330, HDAC9; L-005241 and HDAC11; L-004258. HUVECs were plated in 24-well plates in EGM-2 medium without antibiotics and incubated overnight. The following day, at about 70% confluency, cells were transfected. For each stimulation, siRNA (final concentration 10 nM, except in HDAC9 experiments final concentration = 40 nM) and 1 µl DharmaFECT 4 transfection reagent (Dharmacon) were separately incubated in 50 µl OptiMEM (Invitrogen) for 5 min, thereafter combined and incubated for 20 min and finally diluted in 400 µl EGM-2 without antibiotics before being added to cells. A second transfection was made 24 h later. Forty-eight hours after the second siRNA treatment, the cells were treated with 3 mM VPA or control medium and 24 h later mRNA was extracted and analyzed by real-time PCR to determine target mRNA reduction and t-PA mRNA expression. Results were only used when target reduction was at least 80%. Two negative controls were used for siRNA, in one the DharmaFECT 4 transfection reagent was added alone to cells (mock), in another a control siRNA was used (All Star Negative control, Qiagen). No difference in expression of t-PA or target gene was observed with either control.

### Microarray analysis

Gene expression in VPA-treated and untreated HUVECs from 4 donors was analyzed using the Human Gene 1.0 ST microarray (Affymetrix). Target preparation and hybridization to the DNA microarray were performed according to standard Affymetrix protocols at the Uppsala Array Platform (Uppsala, Sweden). Raw data from the arrays are MIAME compliant and have been deposited in the Gene Expression Omnibus (GEO) database (raw data available at: http://www.ncbi.nlm.nih.gov/geo/query/acc.cgi?token=dnglzumweoisshk&acc=GSE23909). Raw data was analyzed using the RMA (robust multi-array average) method implemented in the Affymetrix software Expression Console. Probe sets with a log2 ratio above +1 or below −1 and a significantly changed expression (p<0.05, false discovery rate (FDR) adjusted p-value) were classified as regulated. Hemostasis genes were identified using the Amigo database (http://amigo.geneontology.org). The regulation of these genes, as well as all genes up and down regulated by VPA can be found in table S1.

### Statistics

Data are presented as mean and standard error of the mean. All comparisons are specified in the figure legends and were performed between samples from the same experiment and time-point to check for statistical significance. Unless otherwise stated in figure legends, the statistical evaluation was performed using paired Student's t-test. A p-value of less than 0.05 was considered significant. For analysis of time series data, two-way analysis of variance (ANOVA) for repeated measures was used.

## Results

### Valproic acid induces t-PA mRNA expression and protein secretion

**Figure 1:**
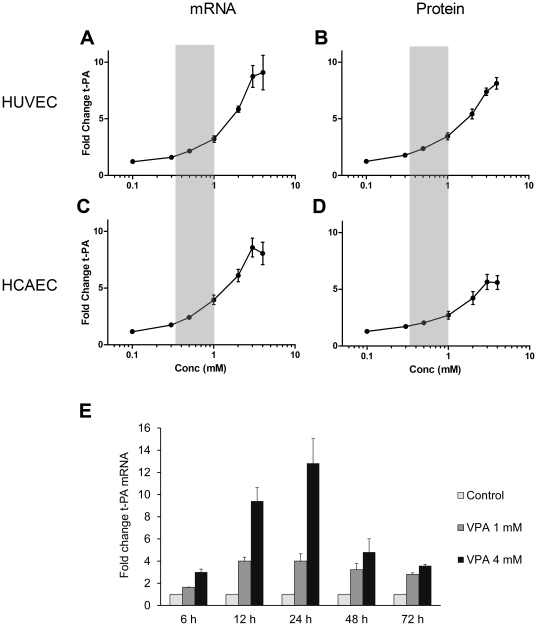
Effects of valproic acid (VPA) on mRNA and protein expression in HUVEC and HCAEC. HUVEC (**A** and **B**) and HCAEC (**C** and **D**) were exposed to different concentrations (0.1–4 mM) of VPA for 24 h. mRNA (**A** and **C**) was quantified with real-time PCR and secreted t-PA protein (**B** and **D**) in conditioned media by ELISA. Values are expressed as fold change over control cells. The shaded areas show the plasma concentration range of VPA achieved after clinical VPA treatment. **E**. HUVECs were treated with 1 mM or 4 mM of VPA and t-PA mRNA quantified after 6, 12, 24, 48 and 72 h. The results show mean values ± SEM of three independent experiments (*n* = 3) performed in duplicate. For Fig. 1E statistical significance was tested using 2-way ANOVA for repeated measures: p(dose)<0.01, p(time)<0.01 and p(dose×time)<0.001.

Incubation of HUVEC cultures with VPA for 24 h caused a significant concentration-dependent increase in t-PA mRNA expression, evident already at concentrations as low as 0.3 mM (p<0.05) and reaching a maximum at 3–4 mM (Fig. 1A). At the maximal concentrations, t-PA mRNA levels were increased 9-fold after 24 h. The levels of t-PA protein released into the medium during VPA stimulation were comparably (8-fold) induced (Fig. 1B). There was a somewhat less pronounced effect of VPA on t-PA secretion during the second 24 h-period of culture (48 h) but maximal protein induction was still maintained on an approximately 5-fold increase (data not shown).

To verify that this effect of VPA was present also in a more representative endothelial cell type, we incubated HCAEC with VPA and measured t-PA mRNA expression (Fig. 1C) and protein secretion (Fig. 1D). A comparable response pattern as in HUVEC could be observed in this cell type after 24 h, with an 8- and 5-fold increase in mRNA and protein, respectively. Similar mRNA and protein levels could also be detected for the time period 24–48 h (data not shown).

In order to investigate the temporal response pattern we exposed HUVECs to a clinically relevant concentration (1 mM) as well as to the maximum *in vitro* tolerated concentration (4 mM) of VPA for up to 72 h and measured t-PA mRNA levels (Fig. 1E). For the 1 mM dose, a ∼4-fold steady-state induction was reached after 12 h and this level of induction remained throughout the period studied (<72 h). The response pattern for the higher dose (4 mM) was somewhat different with an initial transient peak induction of about 12-fold at 24 h, which gradually leveled-off to about 4-fold at 72 h.

**Figure 2:**
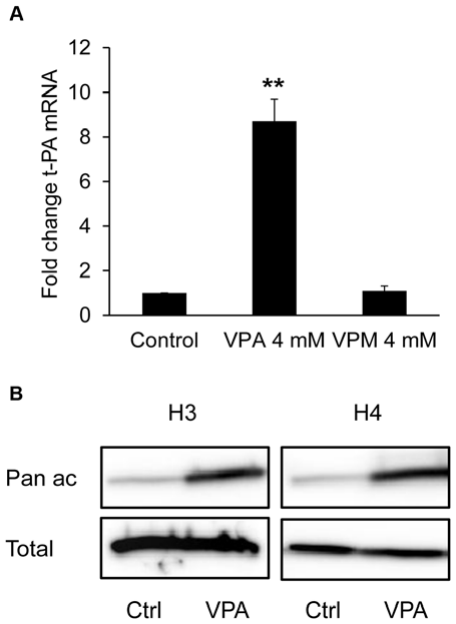
Dependence of HDAC-inhibitory activity for t-PA induction and effect of VPA on global histone acetylation. **A.** t-PA mRNA expression in HUVECs stimulated with 4 mM VPA or 4 mM VPM (a structural amide analogue of VPA which is reported to lack HDAC inhibitory activity) for 24 h. The results are presented as mean values ± SEM of five independent experiments performed in duplicate. **p<0.01. **B.** HUVECs were exposed to 3 mM VPA for 24 h after which cells were harvested in Laemmli sample buffer for analysis of global acetylation of histone H3 and H4 by Western blotting. Data are representative of three independent experiments.

We also compared the effect of VPA (2-propylpentanoic acid) on t-PA expression to that of valpromide (2-propylpentanamide), a structural amide analogue of VPA reported to lack HDAC inhibitory activity [14], [19]. Whereas 4 mM of VPA increased t-PA levels 9-fold, the same concentration of valpromide had no effect on expression of t-PA mRNA in HUVECs (Fig. 2A). Moreover, we confirmed that the t-PA induction by VPA is not due to altered pH, as pH was identical (7.28) in EGM-2 medium with/without 3 mM VPA after over-night incubation at 37°C, 5% CO_2_.

### Valproic acid increases acetylation of histones H3 and H4

To confirm that VPA acts as an HDAC inhibitor in endothelial cells we performed western blot assays with antibodies to acetylated as well as to total histone H3 and H4. These experiments showed an increase of global acetylated histone H3 and H4 after VPA treatment, whereas no induction of total histone proteins could be detected (Fig. 2B).

**Figure 3:**
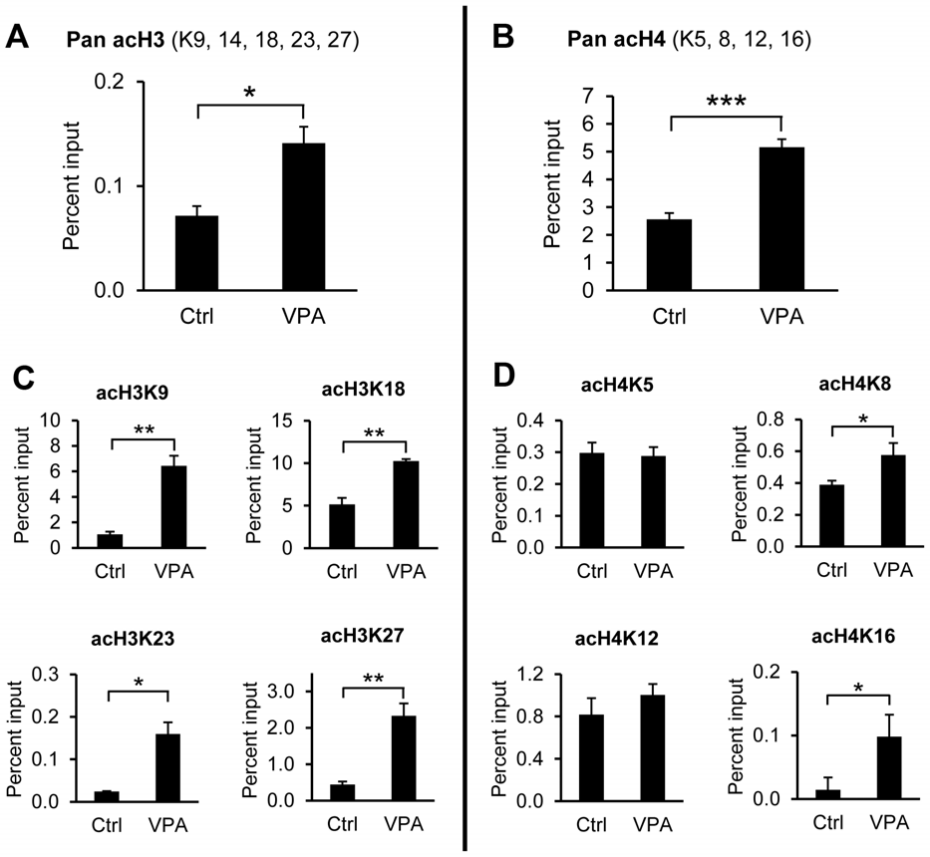
Chromatin Immunoprecipitation (ChIP) for acetylated histones in the t-PA promoter. HUVECs were exposed to 3 mM of VPA for 24 h after which cells were fixed and chromatin harvested. ChIP-analyses for acetylated histone H3 and H4 were performed with real-time PCR primers flanking the major t-PA transcription start site. Data are presented as percent input corrected for background binding and mean values ± SEM of four to five independent experiments are shown. **A.** ChIP for pan-acetylated histone H3. *n* = 4. **B.** ChIP for pan-acetylated H4. *n* = 5. **C.** ChIP for specific histone H3 acetylation. Antibodies for monoacetylated acH3K9, acH3K18, acH3K23 and acH3K27 were used. *n* = 4. **D.** ChIP for specific histone H4 acetylation. Antibodies for monoacetylated acH4K5, acH4K8, acH4K12 and acH4K16 were used. *n* = 5. *p<0.05, **p<0.01, *** p<0.001.

To investigate if VPA increases histone acetylation specifically at the t-PA gene regulatory region, ChIP-analyses were performed using antibodies to pan-acetylated histone H3 and H4, and primers flanking the major t-PA transcription initiation site. This showed a significant 2-fold increase of both acetylated H3 and H4 associated with the region surrounding the major t-PA transcription start site after VPA treatment (Fig. 3A and B). We went on to examine which specific lysine residues in histone H3 and H4 that were acetylated after VPA treatment. Antibodies for specific lysine modifications were also used in ChIP assays and demonstrated a significant increase of acetylation of lysines 9, 18, 23 and 27 on histone H3 as well as lysines 8 and 16 on histone H4 (Fig. 3C and D). Acetylation of H3K14 was undetectable in this system, whereas H4K5 and K12 acetylation was not significantly changed after VPA treatment.

### siRNA knock-down of class I, IIa and IV HDACs does not block t-PA induction by VPA

**Figure 4:**
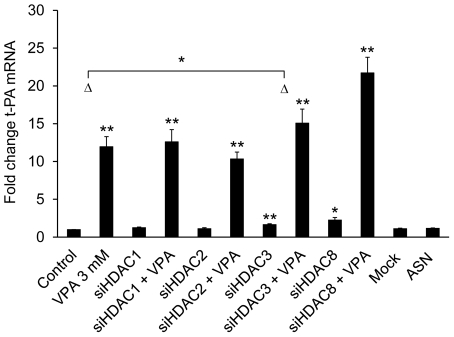
siRNA mediated knock-down of class I HDACs. t-PA mRNA expression in HUVECs treated with class I HDAC-targeting siRNAs for 72 h and then stimulated with 3 mM VPA for 24 h. Mock indicate cells treated with DharmaFECT 4 transfection reagent alone and ASN indicate cells transfected with All Star Negative control siRNA (Qiagen). Unless indicated in the figure, statistical comparisons are made relative to untreated control cells. *p<0.05, **p<0.01. The results are presented as mean values ± SEM of four independent experiments performed in duplicate.

**Figure 5:**
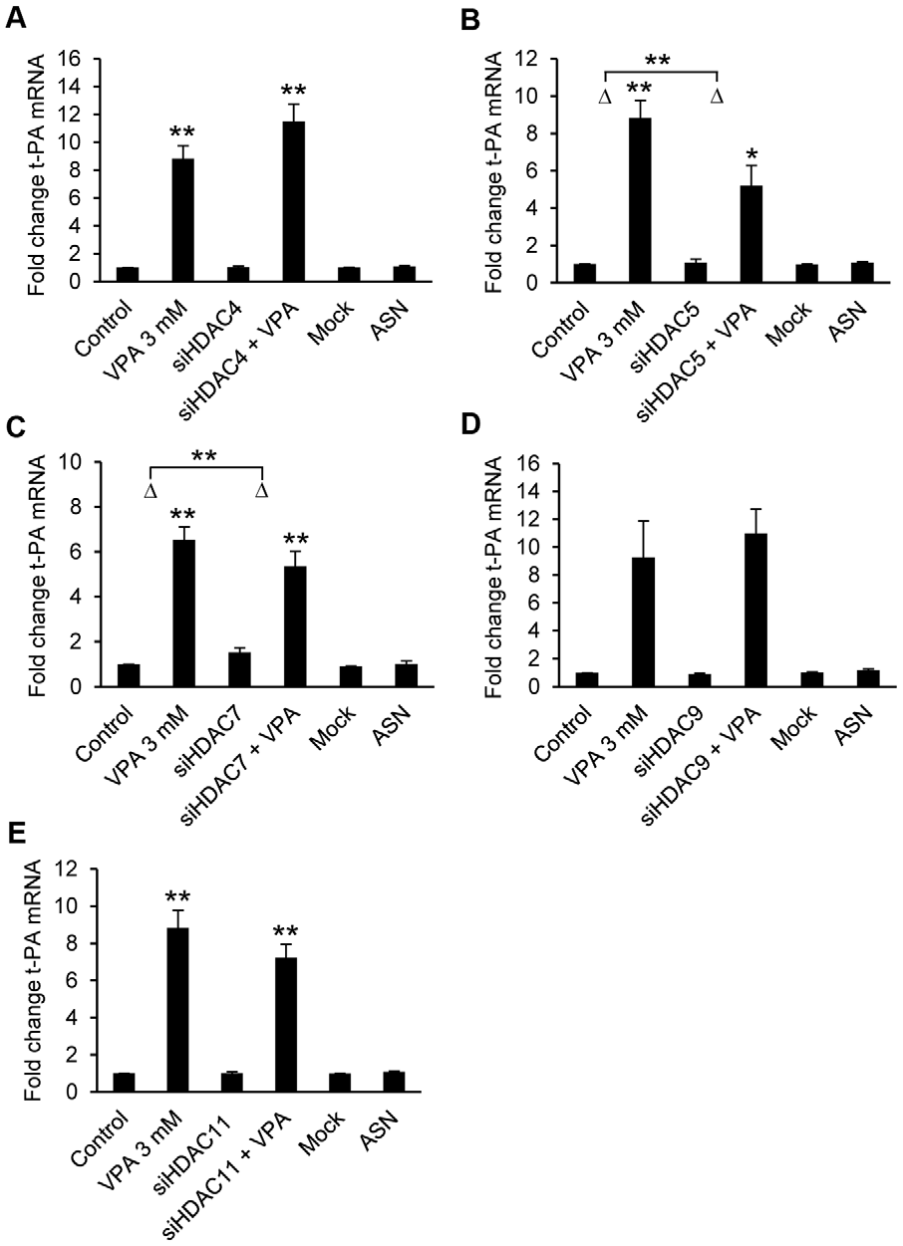
siRNA mediated knock-down of class IIa and class IV HDACs. t-PA mRNA expression in HUVECs treated with **A.** HDAC4, **B.** HDAC5, **C.** HDAC7, **D.** HDAC9 and **E.** HDAC11-targeting siRNA for 72 h and then stimulated with 3 mM VPA for 24 h. Mock indicate cells treated with DharmaFECT 4 transfection reagent alone and ASN indicate cells transfected with All Star Negative control siRNA (Qiagen). Unless indicated in the figure, statistical comparisons are made relative to untreated control cells. *p<0.05, **p<0.01. The results are presented as mean values ± SEM of four independent experiments performed in duplicate (HDAC9, n = 2).

As VPA has been reported to selectively inhibit class I and IIa HDACs, each HDAC enzyme in these two groups was independently depleted with siRNA to identify its potential role in basal as well as VPA-induced t-PA gene expression (Fig. 4 and 5). HDAC11, the only class IV HDAC, was also included in these studies. Treatment of cells with HDAC1, HDAC2, HDAC4, HDAC5, HDAC7, HDAC9 and HDAC11 siRNA had no effect on basal t-PA mRNA expression. However, a modest induction of t-PA mRNA was observed in HDAC3 (67%, p<0.01) and HDAC8 (225%, p<0.05) depleted cells, compared to untreated controls.

Single depletion of any of the 9 tested HDACs was not sufficient to block the VPA-response, as all cells challenged with VPA showed significant inductions of t-PA (p<0.05 or below compared to untreated controls, as well as respective siRNA treated control). However, the magnitude of the t-PA induction was partially reduced with HDAC3, HDAC5 and HDAC7 siRNA treatments. HDAC3 depleted cells showed a modest reduction from 12-fold induction of t-PA in untreated control cells to 9-fold (p<0.05). In HDAC5 and HDAC7 siRNA treated cells the VPA-response was more profoundly weakened; for HDAC5 from 9-fold to 5-fold and for HDAC7 from 6.5-fold to 3.5 fold (both p<0.01).

### Valproic acid causes a selective stimulation of gene expression in endothelial cells

**Figure 6:**
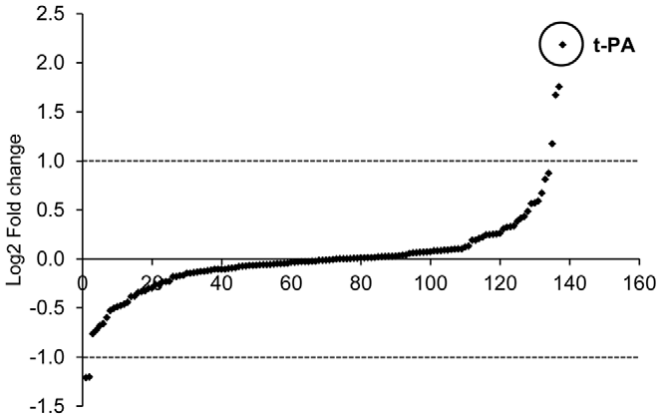
Effect of VPA on the mRNA expression of haemostatic genes. HUVECs (from four individuals) were stimulated with 4 mM VPA for 24 h and mRNA from treated and untreated cells were analyzed using the Human Gene 1.0 ST microarray. Haemostasis genes (a total of 138 genes) were identified using the Amigo database and plotted in the order of increasing log2 fold change. Probe sets with a log2 ratio above +1 or below −1 and a significantly changed expression (p<0.05, FDR adjusted p-value) were classified as regulated. The dot representing t-PA is highlighted (log Fold Change 2.18). The other regulated haemostasis genes are: Tissue factor pathway inhibitor 2 (TFPI2) (log Fold Change 1.18), signal peptide CUB domain EGF-like 1 (SCUBE1) (log Fold Change 1.67), coagulation factor II (thrombin) receptor-like 2 (F2RL2) (log Fold Change 1.75), P-selectin (log Fold Change −1.21) and fermitin family homolog 3 (drosophila) (FERMT3) (log Fold Change −1.20).

In order to determine the effect of high dose VPA on global gene expression, microarray analysis was performed on HUVEC cells after exposure to maximal concentration (4 mM) of VPA for 24 h. On a global scale, a relatively small number of genes, 2.6%, were affected by this treatment. Out of these, not all were up-regulated, but 37% were actually suppressed by the treatment. When looking more specifically at genes involved in hemostatic pathways, the expression levels of the majority of transcripts were not significantly affected by VPA (Fig. 6). Of the 138 genes annotated as being involved in hemostasis (131 unique genes and 7 with 2 probe sets), only 4 genes were up-regulated and 2 genes suppressed. Indeed, the hemostatic gene that was most strongly regulated by VPA was t-PA.

The relative lack of effect of VPA on plasminogen activator inhibitor-1 (PAI-1), urokinase plasminogen activator (u-PA), and von Willebrand (vWF) factor in the array was confirmed by real-time PCR. Maximal doses of VPA only caused a minor, approximately 30%, increase of both PAI-1 and u-PA, and a 30% decrease of vWF transcript (data not shown).

## Discussion

In search of a clinically eligible pharmacological tool to enhance or restore an impaired endogenous fibrinolysis, previous studies have mainly focused on the transcriptional regulation of t-PA via classic transcription factor/cis-element interactions. However, two studies by Kooistra *el al* [9], [10] and a recent study by the group of Kruithof [11] have suggested that the t-PA gene could be sensitive to changes in histone acetylation status. In light of these findings we investigated whether the putative HDAC-inhibitory activity of valproic acid (VPA) [13], [14], a clinically used and generally well-tolerated anti-epileptic drug, could enhance t-PA synthesis in human endothelial cells. We found that VPA markedly increased t-PA mRNA expression and protein secretion in two different endothelial cell types with a dose-dependent response. Importantly, this effect was evident at low, clinically relevant concentrations with a significant increase already at 0.3 mM, and reaching a maximum of approximately 10-fold induction.

Next, we investigated if this profound effect of VPA on t-PA production in endothelial cells was indeed related to the ability of the drug to modify histone acetylation status. Western blot analysis of VPA-treated HUVECs showed increased total histone acetylation, confirming that VPA functions as an HDAC inhibitor in this cell type. Moreover, the VPA analogue valpromide (VPM), a substance lacking HDACi activity [14], [19], failed to induce t-PA in endothelial cells. To determine whether VPA affects acetylation status of histones associated specifically with the t-PA promoter we performed ChIP analyses with antibodies directed against acetylated histone H3 and H4, and primers spanning the major transcription start site. Using pan-acetyl H3 and H4 antibodies, detecting acetylation at a number of different lysines, we observed a significant increase of acetylated histones associated with the t-PA promoter after VPA treatment, further supporting the hypothesis that it is the HDACi activity of VPA that specifically causes the increase in transcription. This is also in accordance with the recently published study by Kruithof and coworkers who noted increased acetylation of histones associated with the t-PA regulatory region 1 kb upstream of the major transcription start site after treatment of HUVEC with the HDACi TSA or MS-275 [11].

Recent accumulating evidence points to the existence of a histone code that is recognized and interpreted by effector proteins with chromatin modifying activities [20]. There are also data implying that certain specific modifications directly influence higher-order chromatin structure and compaction. To confirm the results from the pan-acetyl antibody ChIPs and to get a more detailed view of the acetylation code surrounding the t-PA transcription start site, we looked more specifically at individual lysine acetylation in this region using lysine-specific antibodies. We found that the majority of the H3 lysines covered by the pan-acetyl antibody (K9, 14, 18, 23, and 27) were differentially acetylated after VPA stimulation. For H4, we could detect increased acetylation at two out of four potential acetylation sites, namely K8 and K16. Acetylation of H4K16 specifically has been reported to have a strong influence on higher-order chromatin structure [21]. Interestingly, this was one of the histone acetylation marks at the t-PA promoter that was most noticeably induced after VPA stimulation, perhaps indicating that compaction of chromatin in this area might be reduced by VPA treatment. Taken together these results support the hypothesis that it is indeed the VPA HDAC inhibitory property *per se* that stimulates t-PA production.

Given that the HDACi function of VPA seems important for the regulation of t-PA we wanted to determine the role of the individual HDACs in regulation of t-PA both constitutively and in the VPA response. VPA was initially suggested to be a HDAC class I selective inhibitor [13]. More recent data suggest that VPA inhibits HDACs of both the class I and class II subfamilies (IC_50_ approximately 1 mM), only discriminating against the class IIb HDACs (HDAC6 and HDAC10) which are not inhibited [22]. However, Dunoyer-Geindre *et al* has recently implied the importance of the class I HDACs in the regulation of t-PA [11]. To extend this study and to test the potential dependence of each single HDAC, we performed siRNA knockdown of the class I (HDAC1, 2, 3, and 8), IIa (HDAC4, 5, 7, and 9) and class IV (HDAC11) HDACs and investigated the effect of VPA in these HDAC deficient cells. Although we observed a weakened VPA-response with HDAC3, HDAC5 and HDAC7 depletion, these experiments showed that no single HDAC of class I, IIa and IV is crucial for the induction of t-PA by VPA. It is therefore likely that these factors are redundant and in order to block the effect of VPA, simultaneous knock-down of several HDACs might be required.

Considering that VPA increased global histone acetylation in endothelial cells and acetylation is regarded as a permissive modification, it is possible that hyperacetylation might have caused a generalized increase in gene expression, and that, accordingly, the induction of t-PA expression may be a non-specific effect. To clarify this issue we performed transcription profiling on HUVECs treated with VPA for 24 h, assaying the effect of VPA on approximately 28,000 human genes. Somewhat unexpectedly, the effect of the treatment on global gene expression was quite limited. Only a total of 2.6% of all genes were significantly modified. Out of these, 37% were actually suppressed indicating that HDAC inhibition is not always a stimulatory mechanism but may also suppress gene expression, perhaps via up-regulation of regulatory proteins that are repressors of transcription or mediators of RNA instability [23]. In line with our findings, previous array studies with other HDAC inhibitors have indicated that only a small number of genes, about 2–5%, are in fact affected by HDAC inhibitors [24], [25] in various cell types.

In further support of our hypothesis that the t-PA gene is particularly sensitive to histone acetylation, out of the 138 hemostasis-related genes identified in the AMIGO database, we found that the most powerfully regulated gene was in fact t-PA. The related fibrinolytic gene u-PA and the main inhibitor of t-PA, PAI-1, were only marginally affected by the treatment, which was also confirmed by real-time PCR. Of note, it has been suggested that genes that are dependent on the Sp1 transcription factor for transcription (TATA-less genes) are often negatively regulated by HDACs and greatly induced by HDAC-inhibitors [26], [27], [28], [29]. Interestingly, t-PA belongs to this group of genes, as its promoter contains three Sp1 binding GC-boxes reported to be crucial for constitutive t-PA expression [30], [31]. Sp1 has been shown to recruit cofactors with both HAT and HDAC activities [32], [33], [34] and it is possible that HDAC inhibition affects the balance of these activities in this region hence changing gene expression. As HDACs also are known to de-acetylate many non-histone proteins including Sp1, it is also possible that HDAC inhibition results in acetylation of the Sp1 protein itself potentially changing its DNA binding affinity or protein-protein interactions [34].

Even though the data presented here show an effect of VPA on t-PA production *in vitro*, it remains to be determined whether VPA treatment could be used clinically for stimulation of endogenous fibrinolytic capacity *in vivo*. VPA has been extensively used in epilepsy treatment and its profile of adverse effects is well known, the majority of side effects are mild, reversible, and occurring mainly at high plasma concentrations [35]. More severe adverse effects may occur in children (hepatotoxicity) and pregnant women (teratogenicity), but these groups are rarely considered for cardiovascular prevention. Interestingly, concentrations comparable to those in the lower therapeutic plasma concentration range (0.3–0.5 mM) caused a significant 2–3 fold increase of t-PA synthesis. Unfortunately, endothelial t-PA release is difficult to assess *in vivo* since it requires arterial cannulation with local organ measurement due to the rapid degradation of t-PA by the liver with highly variable clearances rates [36]. If, however, a similar enhancement of t-PA production could be obtained in patients treated with valproic acid it could theoretically reduce the risk of acute atherothrombotic disorders. Indeed, Olesen *et al* recently reported a 40% reduced risk of myocardial infarction in a Danish nation-wide study of epileptic patients treated with VPA [37]. Even though VPA has been reported to affect several aspects of the hemostatic system [35], it is conceivable that a substantial part of this reduced risk could be attributable to enhanced t-PA production.

In summary, this is to our knowledge the first time that an already approved and registered pharmacological agent has been shown to potentiate t-PA expression in clinically relevant concentrations *in vitro* and hence VPA may open up an opportunity for pharmacological modulation of the fibrinolytic system *in vivo*.

## Supporting Information

Table S1 **Regulation of global endothelial gene expression by VPA.** The table lists data obtained from microarray experiments on HUVECs treated with 4 mM VPA for 24 h. The table is divided into *genes up-regulated by VPA*, *genes down-regulated by VPA* and a complete list on *regulation of all hemostasis genes by VPA*. Hemostasis genes were identified using the Amigo database (http://amigo.geneontology.org). Presented data are mean of four individual experiments and are listed as fold change compared to untreated control cells.
(XLS)
